# Neurological adverse events of ROS1 inhibitors for non-small cell lung cancer: data from the FDA adverse event reporting system

**DOI:** 10.3389/fneur.2025.1691324

**Published:** 2026-01-08

**Authors:** Xueying Wei, Qianhui Lai, Lingxiao Zheng

**Affiliations:** 1 Department of Geriatrics, The Eighth Affiliated Hospital of Sun Yat-sen University, Shenzhen, Guangdong, China; 2 Department of General Medicine, The Eighth Affiliated Hospital of Sun Yat-sen University, Shenzhen, Guangdong, China

**Keywords:** ROS1 inhibitors, neurological adverse events, FAERS database, safety, non-small cell lung cancer

## Abstract

**Background:**

ROS1 inhibitors play a critical role in the treatment of ROS1 fusion–positive non–small cell lung cancer (NSCLC). Although agents such as Crizotinib, Ceritinib, Lorlatinib, Entrectinib, and Repotrectinib have demonstrated strong efficacy and intracranial activity, their neurological safety profiles remain insufficiently characterized in real-world settings. This study aimed to evaluate the neurological adverse events (AEs) of ROS1 inhibitors using the FDA Adverse Event Reporting System (FAERS) database.

**Methods:**

We conducted a pharmacovigilance analysis of FAERS reports from 2011Q4 to 2024Q4. Disproportionality analysis was used to detect potential AE signals, followed by time-to-onset and logistic regression analyses to assess the onset timing and mortality risk associated with neurological AEs.

**Results:**

A total of 7,296 AE reports related to ROS1 inhibitors were identified. Neurological AEs were prominent, with distinct patterns across drug generations. Common events included dysgeusia, dysarthria, cognitive disorder, and taste disturbance. Novel inhibitors such as Lorlatinib, Entrectinib, and Repotrectinib showed earlier onset of neurotoxicity, whereas older agents (Crizotinib and Ceritinib) were associated with delayed or cumulative neurological events. Despite stronger neurotoxicity signals, Entrectinib demonstrated a relatively favorable safety profile with fewer fatal outcomes.

**Conclusion:**

This study provides real-world evidence that newer ROS1 inhibitors exhibit earlier but generally manageable neurological AEs. Clinicians should implement early neurotoxicity monitoring and individualized risk assessment to ensure safe and effective targeted therapy for ROS1-positive NSCLC.

## Highlights

- The heterogeneous spectrum of post-marketing neurological toxicities for ROS1 inhibitors is largely predictable from preapproval evidence.
- The analysis of individual case safety reports from pharmacovigilance databases supports post-marketing surveillance of adverse events of clinical interest.
- Novel inhibitors (such as Reportinib and Entretinib) tend to occur adverse events relatively early, but exhibits better safety profiles. Long-term use of older agents (such as Crizotinib and Ceritinib) carries a higher risk, even may cause death, deserve prioritization by clinicians.

## 1 Introduction

Lung cancer remains the leading cause of cancer-related deaths worldwide, with Non-Small Cell Lung Cancer (NSCLC) accounting for approximately 85%−90% of all cases. Globally, about 2.2 million new cases and 1.8 million deaths occur each year, making NSCLC a major global health concern (1). ROS1 fusion is an actionable target alteration that occurs in 1%−2% of patients with NSCLC (2). The identification of this target is of great significance, it not only provides a clear molecular typing basis for NSCLC but also serves as a druggable biomarker due to its unique oncogenic mechanism, driving the development of precision medicine in the field of lung cancer. As a therapeutic target, ROS1 fusion proteins can be inhibited by specific tyrosine kinase inhibitors [TKIs; (3)]. The first ROS1 inhibitor, Crizotinib, was approved by the FDA in 2016 for the treatment of ROS1-positive NSCLC (4). In recent years, new-generation ROS1 inhibitors, such as Entretinib, Ceritinib, Lorlatinib, and Reportinib, have emerged (5–8). Compared with the initial Crizotinib, these novel agents exhibit advantages in central nervous system (CNS) penetration, antitumor activity, and resistance overcoming, particularly in better controlling brain metastases (9, 10).

Although ROS1 inhibitors have shown remarkable efficacy in ROS1-positive NSCLC, their neurological safety profiles deserve careful attention. In the CROWN trial, lorlatinib demonstrated superior CNS activity compared with crizotinib but was associated with a higher incidence of neurocognitive and mood-related adverse events (11). Similarly, the TRIDENT-1 study of Reportinib reported frequent neurological toxicities such as dizziness, dysgeusia, and paresthesia (12). These findings indicate that while newer ROS1 inhibitors improve intracranial disease control, CNS-related adverse reactions remain clinically significant. However, evidence from clinical trials is derived from highly selected populations, highlighting the need for real-world pharmacovigilance analyses to comprehensively evaluate neurological safety.

The Food and Drug Administration Adverse Event Reporting System (FAERS) is a comprehensive database that collects and archives adverse event reports from healthcare professionals, patients, and manufacturers. This study aims to utilize the FAERS database to compare the risks of nervous system-related adverse events associated with different ROS1 inhibitors in NSCLC patients and explore their onset timing and potential risk factors influencing patient outcomes, thereby providing valuable reference information for clinical practice.

## 2 Methods

### 2.1 Data source and processing

Data were sourced from the FAERS database, containing adverse event (AE) reports submitted to the US FDA by healthcare professionals, consumers, and manufacturers. The data extraction period covered the fourth quarter of 2011 to the fourth quarter of 2024 and included seven sections: patient demographic (DEMO), drug-related information (DRUG), adverse event data (REAC), reported source information (RPSR), therapeutic start and end dates of the reported drugs (THER), drug usage or diagnostic indications (INDI), and patient outcomes related to adverse events (OUTC). Duplicate records were removed according to the recommended methodology. The fields PRIMARYID, CASEID, and FDA_DT (the submission date of the report to the FDA) from the DEMO file were selected. For cases with duplicate CASEIDs, the record with the most recent FDA_DT or the highest PRIMARYID was retained. Adverse events in the REAC file were standardized using the Medical Dictionary for Regulatory Activities (MedDRA, version 27.0). MedDRA is a hierarchical terminology system composed of multiple levels, starting from the most specific Lower Level Term (LLT) to Preferred Term (PT), High Level Term (HLT), High Level Group Term (HLGT), and finally, System Organ Class (SOC). The standardization process mapped the originally reported adverse event terms to the corresponding standardized MedDRA terms. This study primarily focused on the SOC and PT levels. Quality control procedures included duplicate removal, consistency checks across datasets, and exclusion of incomplete or implausible demographic data (e.g., negative ages or missing dates). All data processing followed FDA and ISPE pharmacovigilance guidelines to minimize bias.

The keywords “Brigatinib”, “Cabozantinib”, “Ceritinib”, “Crizotinib”, “Lorlatinib”, “Entretinib”, and “Reportinib” were used to retrieve relevant drug records from DRUG file. To identify AEs related to non-small cell lung cancer, INDI file was searched using the following terms: “non-small cell lung cancer”, “non-small cell lung cancer metastatic”, “non-small cell lung cancer recurrent”, “non-small cell lung cancer stage iiia”, and “non-small cell lung cancer stage iv”.

### 2.2 Disproportionality analysis

Disproportionality analysis is a cornerstone of pharmacovigilance used to identify potential safety signals by quantifying the disproportionality between the observed and expected frequencies of specific drug–event pairs in the FAERS database. In this study, we employed four widely recognized algorithms—the Reporting Odds Ratio (ROR), Proportional Reporting Ratio (PRR), Bayesian Confidence Propagation Neural Network (BCPNN; Information Component, IC), and Empirical Bayes Geometric Mean [EBGM; (13)]. The use of both frequentist (ROR and PRR) and Bayesian (IC and EBGM) methods enhances the robustness of signal detection and reduces the likelihood of spurious associations arising from random variation. ROR and PRR were selected for their interpretability and high sensitivity in early signal detection, while IC and EBGM provide improved specificity and are better suited for datasets with sparse reports. Signal detection thresholds were defined as follows:ROR: lower limit of the 95% CI (ROR_025) > 1 and case count ≥ 3; PRR: PRR ≥ 2 and χ^2^ ≥ 4; IC: IC_025 > 0; EBGM: EBGM_05 > 2. To account for multiple testing, false discovery rate (FDR) correction was applied using the Benjamini–Hochberg method. The relevant formulas are provided in Supplementary Table 1.

### 2.3 Statistical analysis

Time to onset (TTO) analysis was defined as the interval between the date of the AE (EVENT_DT) and the start date of the drug administration (START_DT). Only cases with complete and accurate date records were included in the analysis. The cumulative curves of TTO in different systems were plotted to observe the differences in TTO. For logistic analysis, first, these seven drugs namely Crizotinib, Entretinib, Lorlatinib and Ceritinib, Brigatinib, Cabozantinib, and Reportinib were included. Crizotinib was set as the reference group for drug comparison. Then, logistic regression analysis was conducted on the systems with positive results of disproportionality analysis to explore the impact of adverse reactions in different systems on the adverse outcome of hospitalization. To investigate factors associated with fatal outcomes (Death), we performed multivariable logistic regression analyses. The independent variables included age, gender, weight, and drug treatment group. Age was categorized into two groups: <65 years and ≥65 years, while weight was divided into three categories: <50, >100 kg, and 50–100 kg. *P*-values less than 0.05 were considered statistically significant. All analyses were conducted using R software (version 4.3.1).

## 3 Results

### 3.1 Baseline characteristics of patients receiving ROS1 inhibitors

A total of 7,296 AE reports related to ROS1 inhibitors were included, covering Crizotinib (*n* = 2974), Ceritinib (*n* = 1272), brigatinib (*n* = 1183), Lorlatinib (*n* = 1129), Entretinib (*n* = 450), Cabozantinib (*n* = 165), and Reportinib (*n* = 123), as shown in Table 1.

**Table 1:** Baseline characteristics of ROS1 inhibitors.

**Characteristics**	**Crizotinib (*n* = 2,974)**	**Cabozantinib (*n* = 165)**	**Ceritinib (*n* = 1,272)**	**Brigatinib (*n* = 1,183)**	**Lorlatinib (*n* = 1,129)**	**Entretinib (*n* = 450)**	**Reportinib (*n* = 123)**
**Gender**							
Male	1,563 (52.6%)	59 (35.8%)	627 (49.3%)	574 (48.5%)	584 (51.7%)	222 (49.3%)	36 (29.3%)
Female	1,213 (40.8%)	61 (37.0%)	561 (44.1%)	442 (37.4%)	448 (39.7%)	181 (40.2%)	73 (59.3%)
Missing	198 (6.7%)	45 (27.3%)	84 (6.6%)	167 (14.1%)	97 (8.6%)	47 (10.4%)	14 (11.4%)
**Age**							
<18	2 (0.1%)	0	1 (0.1%)	38 (3.2%)	6 (0.5%)	51 (11.3%)	8 (6.5%)
18–64	1,309 (44.0%)	46 (27.9%)	665 (52.3%)	363 (30.7%)	554 (49.1%)	127 (28.2%)	44 (35.8%)
≥65	1,253 (38.8%)	54 (32.7%)	301 (23.7%)	262 (22.1%)	323 (28.6%)	123 (27.3%)	37 (30.1%)
Missing	510 (17.1%)	65 (39.4%)	305 (24.0%)	520 (44.0%)	246 (21.8%)	149 (33.1%)	34 (27.6%)
**Weight**							
<50	229 (7.7%)	7 (4.2%)	56 (4.4%)	27 (2.3%)	79 (7.0%)	30 (6.7%)	1 (0.8%)
50–100	892 (30.0%)	48 (29.1%)	277 (21.8%)	195 (16.5%)	335 (29.7%)	121 (26.9%)	2 (1.6%)
>100	56 (1.9%)	5 (3.0%)	12 (0.9%)	10 (0.8%)	30 (2.7%)	1 (0.2%)	5 (4.1%)
Missing	1,797 (60.4%)	105 (63.6%)	927 (72.9%)	951 (80.4%)	685 (60.7%)	298 (66.2%)	115 (93.5%)
**Reporter**							
Consumer	867 (29.2%)	30 (18.2%)	421 (33.1%)	430 (36.3%)	329 (29.1%)	50 (11.1%)	68 (55.3%)
Health professional	92 (3.1%)	14 (8.5%)	69 (5.4%)	57 (4.8%)	129 (11.4%)	33 (7.3%)	16 (13.0%)
Physician	1,474 (49.6%)	113 (68.5%)	510 (40.1%)	582 (49.2%)	572 (50.7%)	330 (73.3%)	18 (14.6%)
Other Professional	350 (11.8%)	5 (3.0%)	189 (14.9%)	21 (1.8%)	28 (2.5%)	2 (0.4%)	0
Pharmacist	182 (6.1%)	3 (1.8%)	32 (2.5%)	84 (7.1%)	57 (5.0%)	34 (7.6%)	17 (13.8%)
Missing	9 (0.3%)		51 (4.0%)	9 (0.8%)	14 (1.3%)	1 (0.2%)	4 (3.3%)
**Outcome**							
Hospitalization	656 (22.1%)	62 (37.6%)	311 (24.4%)	252 (21.3%)	227 (20.1%)	66 (14.7%)	22 (17.9%)
Death	1,011 (34.0%)	37 (22.4%)	447 (35.1%)	274 (23.2%)	310 (27.5%)	64 (14.2%)	16 (13.0%)
Disability	17 (0.6%)	1 (0.6%)	9 (0.7%)	1 (0.1%)	9 (0.8%)	3 (0.7%)	1 (0.8%)
Life-threatening	96 (3.2%)	3 (1.8%)	17 (1.3%)	13 (1.1%)	18 (1.6%)	8 (1.8%)	4 (3.3%)
Other outcomes	597 (20.1%)	13 (7.9%)	355 (27.9%)	547 (46.2%)	357 (31.6%)	150 (33.3%)	24 (19.5%)
Missing	597 (20.1%)	49 (29.7%)	133 (10.5%)	96 (8.1%)	208 (18.4%)	159 (35.3%)	56 (45.5%)

Patient characteristics varied notably across agents. Male predominance was observed for Crizotinib (52.6%) and Lorlatinib (51.7%), while Reportinib showed a female majority (59.3%). Entretinib and Ceritinib exhibited relatively balanced gender distributions. Age group differences were also pronounced. While pediatric cases (<18 years) were rare, Entretinib (11.3%) and brigatinib (3.2%) were more frequently reported in younger populations. In contrast, Crizotinib had the highest proportion of elderly patients (≥65 years, 38.8%). Weight data were largely missing, especially for Reportinib (93.5%) and brigatinib (80.4%), limiting body size assessment. Among available data, most patients fell within the 50–100 kg range, particularly in the Crizotinib and Lorlatinib groups. Reporter profiles highlighted variations in pharmacovigilance channels. Reports by physicians were predominant for most agents, especially Entretinib (73.3%) and Cabozantinib (68.5%). In contrast, over half of the reports for Reportinib (55.3%) originated from consumers, possibly indicating different usage patterns or levels of post-marketing engagement. Clinical outcomes further illustrated the diversity in drug profiles. Death was the most frequently reported outcome for Crizotinib (34.0%) and Ceritinib (35.1%), while brigatinib exhibited a high proportion of “other outcomes” (46.2%), potentially reflecting a broader spectrum of non-serious events. Entretinib and Reportinib had relatively lower death rates (14.2% and 13.0%, respectively), with Reportinib also showing a modest rate of hospitalization (17.9%), possibly reflecting shorter post-approval observation time or different patient selection.

**Figure 1:**
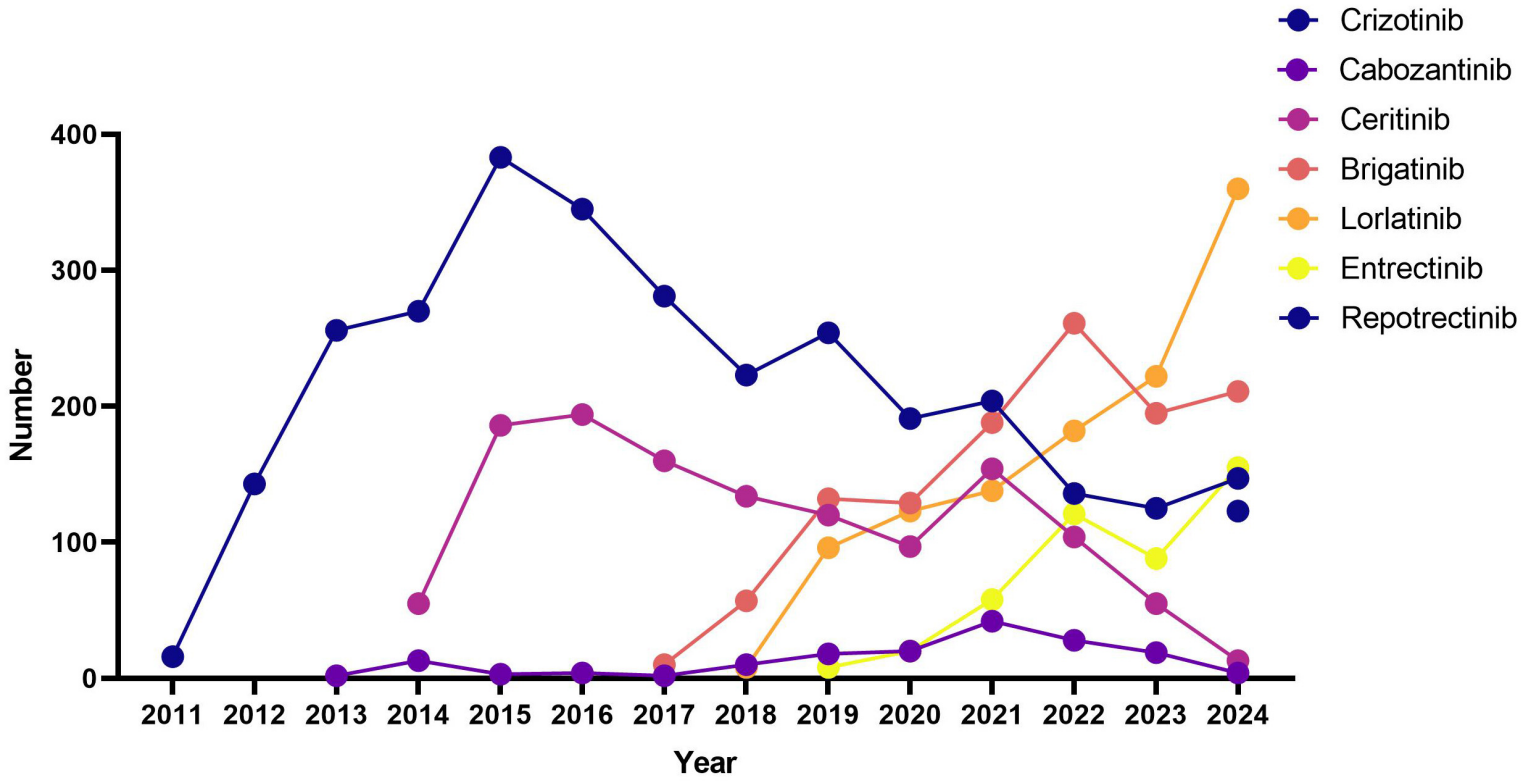
Reporting year of ROS1 inhibitors.

These demographic and clinical differences were consistent with the temporal distribution of AE reports (Figure 1). Crizotinib, the earliest approved ROS1 inhibitor, exhibited a gradual rise in AE reports from 2011, peaking in 2020, followed by a slight decline. Ceritinib entered the database in 2014 and peaked by 2015–2016. Brigatinib and Lorlatinib reports surged after 2017 and 2018, respectively, with Lorlatinib showing a marked increase by 2021. Entretinib and Reportinib demonstrated sharp but late upticks in reporting, consistent with their more recent market introductions. Cabozantinib showed low-level but consistent reporting throughout the years, likely reflecting off-label or niche use.

### 3.2 Comparison of nervous system adverse events at the SOC level

**Figure 2:**
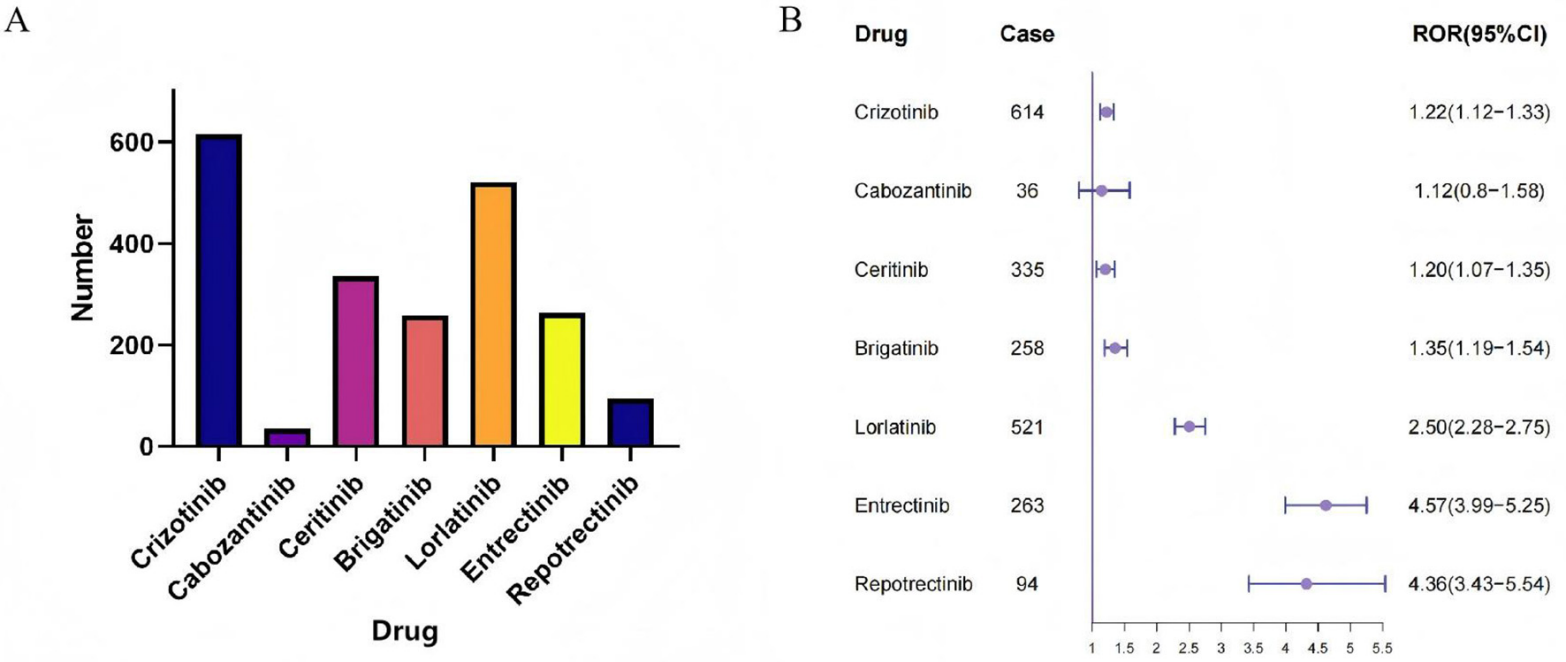
SOC distribution of ROS1 inhibitor. **(A)** The frequency of SOC distribution of ROS1 inhibitor. **(B)** The forest plot of SOC distribution of ROS1 inhibitor.

To explore the neurotoxicity profiles of ROS1 inhibitors, we analyzed the occurrence and reporting odds ratios (RORs) for “Nervous system disorders” at the SOC level (Figure 2). A total of 2,121 nervous system AE reports were identified across agents. Crizotinib, the most frequently reported drug, showed 614 cases with a mild but significant signal (ROR 1.22, 95% CI 1.12–1.33). Ceritinib and Brigatinib demonstrated similar moderate associations (ROR 1.20, 95% CI 1.07–1.35; and ROR 1.35, 95% CI 1.19–1.54, respectively). Lorlatinib exhibited a stronger signal (ROR 2.50, 95% CI 2.28–2.75), while Entretinib and Reportinib showed the highest disproportionality (ROR 4.57, 95% CI 3.99–5.25; and ROR 4.36, 95% CI 3.43–5.54, respectively). Cabozantinib presented the weakest association (ROR 1.12, 95% CI 0.80–1.58) with only 36 reports.

Overall, SOC-level analysis revealed substantial heterogeneity in neurotoxicity signals, with newer-generation inhibitors (Lorlatinib, Entretinib, and Reportinib) exhibiting stronger associations than earlier agents such as Crizotinib or Cabozantinib.

### 3.3 Comparative analysis of nervous system adverse events at the PT level

**Figure 3:**
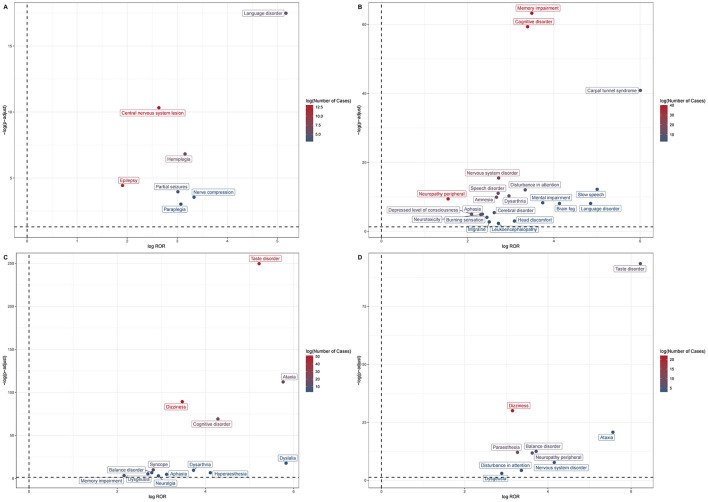
Volcano plots illustrating the distribution of Preferred Term (PT)–level nervous system adverse events associated with ROS1 inhibitors. **(A)** Ceritinib; **(B)** Lorlatinib; **(C)** Entretinib; **(D)** Reportinib. Each point represents an individual PT, where the x-axis denotes the log10(ROR) and the y-axis denotes the –log10(P value). Dots above the horizontal significance threshold line indicate statistically significant signals.

At the PT level, marked heterogeneity was observed among ROS1 inhibitors (Table 2, Figure 3). Several AEs were shared across multiple agents, indicating potential class effects, whereas others were drug-specific.

**Table 2:** Nervous system AEs at the PT level of ROS1 inhibitors.

**Drug**	**PT**	**N**	**ROR (95% Cl)**	**PRR (χ^2^)**	**EBGM (EBGM05)**	**IC (IC025)**
Crizotinib	Dysgeusia	57	5.7 (4.29–7.58)	5.68 (184.52)	4.92 (3.88)	2.3 (1.89)
	Ageusia	14	5.7 (3.21–10.1)	5.69 (45.43)	4.94 (3.06)	2.3 (1.5)
Cabozantinib	Taste disorder	4	8.99 (3.33–24.25)	8.93 (27.67)	8.78 (3.83)	3.13 (1.82)
	Syncope	3	3.88 (1.24–12.11)	3.86 (6.32)	3.84 (1.48)	1.94 (0.49)
Ceritinib	Central nervous system lesion	13	6.2 (3.48–11.03)	6.18 (50.34)	5.62 (3.47)	2.49 (1.67)
	Epilepsy	11	3.75 (2.03–6.93)	3.75 (20.64)	3.56 (2.13)	1.83 (0.97)
	Hemiplegia	6	8.91 (3.74–21.23)	8.9 (35.76)	7.71 (3.73)	2.95 (1.77)
	Language disorder	5	36.05 (11.44–113.62)	36.01 (99.29)	21.43 (8.2)	4.42 (3)
	Partial seizures	4	8.07 (2.81–23.2)	8.07 (21.35)	7.09 (2.93)	2.83 (1.43)
	Paraplegia	3	8.41 (2.48–28.55)	8.4 (16.77)	7.35 (2.64)	2.88 (1.31)
	Nerve compression	3	10.09 (2.92–34.86)	10.08 (20.46)	8.57 (3.04)	3.1 (1.51)
Brigatinib	Somnolence	16	4.36 (2.63–7.25)	4.35 (38.64)	4.13 (2.7)	2.05 (1.32)
	Memory impairment	14	3.88 (2.26–6.67)	3.87 (28.13)	3.71 (2.36)	1.89 (1.12)
	Subarachnoid hemorrhage	3	8.2 (2.46–27.31)	8.19 (16.75)	7.36 (2.69)	2.88 (1.33)
Lorlatinib	Cognitive disorder	40	10.49 (7.44–14.79)	10.4 (279.69)	8.73 (6.55)	3.13 (2.63)
	Memory impairment	40	11.22 (7.94–15.84)	11.12 (299.52)	9.22 (6.91)	3.2 (2.71)
	Neuropathy peripheral	36	2.92 (2.08–4.09)	2.9 (42.4)	2.79 (2.1)	1.48 (0.99)
	Nervous system disorder	18	6.58 (4.02–10.78)	6.56 (74.67)	5.89 (3.9)	2.56 (1.85)
	Speech disorder	13	6.53 (3.66–11.66)	6.51 (53.45)	5.86 (3.6)	2.55 (1.73)
	Amnesia	12	6.36 (3.48–11.62)	6.34 (47.73)	5.72 (3.45)	2.52 (1.67)
	Depressed level of consciousness	10	4.26 (2.23–8.15)	4.26 (22.9)	3.99 (2.32)	2 (1.09)
	Dysarthria	10	7.77 (3.98–15.17)	7.76 (50.7)	6.82 (3.9)	2.77 (1.84)
	Disturbance in attention	9	10.09 (4.91–20.7)	10.07 (60.78)	8.5 (4.66)	3.09 (2.09)
	Aphasia	8	5.07 (2.45–10.51)	5.06 (23.61)	4.68 (2.54)	2.23 (1.21)
	Carpal tunnel syndrome	8	64.25 (22.28–185.25)	64.12 (213.06)	28.05 (11.57)	4.81 (3.59)
	Neurotoxicity	8	4.94 (2.38–10.23)	4.93 (22.76)	4.57 (2.48)	2.19 (1.18)
	Cerebral disorder	7	6.13 (2.79–13.47)	6.12 (26.61)	5.54 (2.87)	2.47 (1.39)
	Burning sensation	6	5.45 (2.34–12.69)	5.44 (19.56)	4.99 (2.46)	2.32 (1.17)
	Mental impairment	5	13.37 (4.96–36.04)	13.36 (44.75)	10.67 (4.66)	3.42 (2.1)
	Slow speech	4	32.09 (9.05–113.77)	32.06 (72.23)	19.64 (6.81)	4.3 (2.74)
	Migraine	4	5.66 (2.01–15.96)	5.66 (13.73)	5.17 (2.17)	2.37 (1)
	Brain fog	4	17.5 (5.57–55)	17.49 (45.6)	13.09 (5.02)	3.71 (2.23)
	Leukoencephalopathy	3	6.56 (1.96–21.93)	6.56 (12.44)	5.89 (2.15)	2.56 (1.01)
	Language disorder	3	28.88 (6.9–120.87)	28.86 (50.42)	18.41 (5.56)	4.2 (2.47)
	Head discomfort	3	8.49 (2.49–28.99)	8.49 (16.84)	7.36 (2.64)	2.88 (1.31)
Entretinib	Dizziness	51	11.11 (8.3–14.86)	10.7 (416.4)	9.97 (7.82)	3.32 (2.89)
	Taste disorder	44	37.15 (26.5–52.09)	35.89 (1,174.97)	28.44 (21.43)	4.83 (4.35)
	Cognitive disorder	22	19.45 (12.39–30.52)	19.12 (330.45)	16.83 (11.55)	4.07 (3.43)
	Ataxia	15	54.2 (29.67–99.01)	53.57 (550.74)	38.4 (23.2)	5.26 (4.44)
	Syncope	10	7.04 (3.72–13.33)	6.99 (48.82)	6.69 (3.92)	2.74 (1.84)
	Balance disorder	7	6.88 (3.21 - 14.75)	6.85 (33.28)	6.56 (3.47)	2.71 (1.66)
	Memory impairment	6	4.45 (1.97–10.05)	4.43 (15.43)	4.32 (2.18)	2.11 (1)
	Dysgeusia	6	6.47 (2.85–14.71)	6.45 (26.34)	6.19 (3.11)	2.63 (1.51)
	Dysarthria	5	13.26 (5.28–33.31)	13.21 (51.33)	12.1 (5.6)	3.6 (2.36)
	Aphasia	4	8.69 (3.15–23.93)	8.66 (25.46)	8.19 (3.51)	3.03 (1.69)
	Neuralgia	3	7.64 (2.38–24.49)	7.62 (16.33)	7.26 (2.74)	2.86 (1.36)
	Dyslalia	3	56.76 (14.66–219.75)	56.63 (114.76)	39.94 (12.87)	5.32 (3.63)
	Hyperaesthesia	3	17.27 (5.18–57.6)	17.23 (40.59)	15.36 (5.61)	3.94 (2.39)
Reportinib	Dizziness	22	8.87 (5.75–13.67)	8.34 (143.26)	8.34 (5.81)	3.06 (2.44)
	Paraesthesia	8	9.64 (4.78–19.44)	9.43 (60.42)	9.43 (5.24)	3.24 (2.26)
	Taste disorder	7	74.66 (35.3–157.9)	73.09 (497.66)	73.06 (39.04)	6.19 (5.16)
	Balance disorder	6	13.16 (5.87–29.5)	12.93 (66.15)	12.93 (6.58)	3.69 (2.59)
	Neuropathy peripheral	6	12.33 (5.5–27.65)	12.12 (61.32)	12.12 (6.17)	3.6 (2.5)
	Dysgeusia	3	7.42 (2.38–23.13)	7.36 (16.52)	7.36 (2.84)	2.88 (1.43)
	Nervous system disorder	3	17.8 (5.71–55.48)	17.65 (47.13)	17.64 (6.82)	4.14 (2.69)
	Ataxia	3	47.28 (15.17–147.37)	46.85 (134.61)	46.84 (18.09)	5.55 (4.1)
	Disturbance in attention	3	10.29 (3.3–32.08)	10.21 (24.94)	10.21 (3.94)	3.35 (1.9)

Sensory disturbances: Dysgeusia was frequently reported across Crizotinib (*N* = 57, ROR 5.70, 95% CI 4.29–7.58), Entretinib (*N* = 6, ROR 6.47, 95% CI 2.85–14.71), and Reportinib (*N* = 3, ROR 7.42, 95% CI 2.38–23.13), suggesting a recurrent sensory signature.

Cognitive and memory impairment: Lorlatinib showed the strongest associations, including memory impairment (*N* = 40, ROR 11.22) and cognitive disorder (*N* = 40, ROR 10.49), while Entretinib also demonstrated high signals for cognitive disorder (*N* = 22, ROR 19.45). Memory impairment was additionally observed for Brigatinib (*N* = 14, ROR 3.88) and Entretinib (*N* = 6, ROR 4.45).

Motor and coordination disorders: Entretinib and Reportinib exhibited the most pronounced motor-related AEs, including ataxia (*N* = 15, ROR 54.20; *N* = 3, ROR 47.28) and dysarthria (*N* = 5, ROR 13.26), reflecting their strong CNS activity.

Drug-specific findings: Lorlatinib demonstrated the broadest neurotoxicity spectrum, including rare but intense signals such as brain fog (*N* = 4, ROR 17.50), carpal tunnel syndrome (*N* = 8, ROR 64.25), language disorder (*N* = 3, ROR 28.88), and slow speech (*N* = 4, ROR 32.09). Entretinib also exhibited high RORs for taste disorder (*N* = 44, ROR 37.15) and dizziness (*N* = 51, ROR 11.11). Reportinib, despite fewer reports, showed remarkable RORs for taste disorder (*N* = 7, ROR 74.66), peripheral neuropathy (*N* = 6, ROR 12.33), and attention disturbance (*N* = 3, ROR 10.29). Ceritinib showed distinctive associations with hemiplegia (*N* = 6, ROR 8.91) and language disorder (*N* = 5, ROR 36.05). Brigatinib was mainly linked to somnolence (*N* = 16, ROR 4.36) and subarachnoid hemorrhage (*N* = 3, ROR 8.20), whereas Cabozantinib reported only a few mild neuro-AEs without strong signals.

Collectively, dysgeusia, memory impairment, and cognitive disorder were recurrent across several agents, whereas Lorlatinib, Entretinib, and Reportinib demonstrated the most intense and diverse neurotoxicity profiles.

### 3.4 Time-to-onset comparison of nervous system adverse events

**Figure 4:**
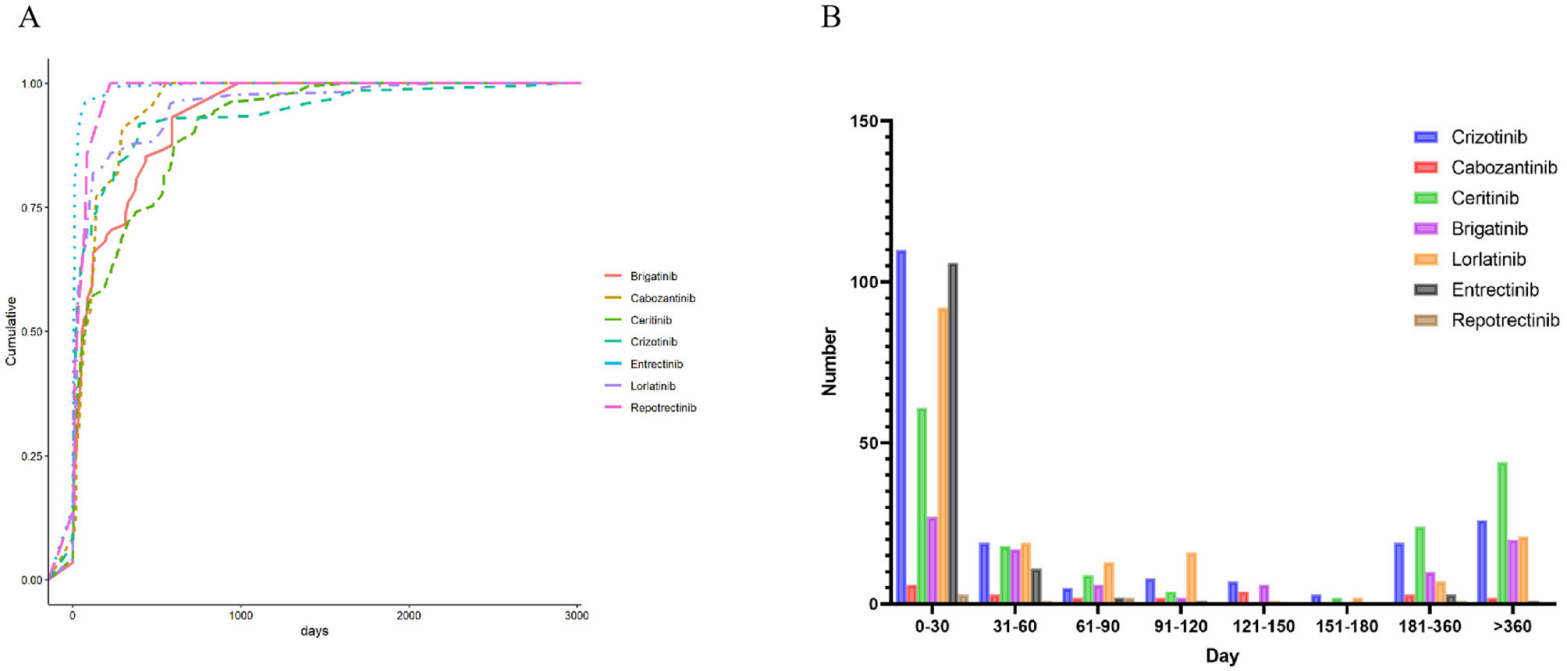
Time-to-onset analysis of nervous system adverse events among patients receiving ROS1 inhibitors. **(A)** Cumulative curves showing the proportion of events over time from treatment initiation. **(B)** Frequency distribution histogram illustrating the onset timing (in days) for each drug group.

TTO analysis revealed distinct latency patterns among ROS1 inhibitors (Figure 4). Entretinib and Lorlatinib displayed rapid-onset neurotoxicity, with most AEs occurring within the first 30 days of treatment (*N* = 106 and *N* = 92, respectively). Reportinib showed a similar early-onset pattern (*N* = 3 within 30 days), though the total number of reports remained limited. In contrast, Crizotinib, and Ceritinib exhibited broader and delayed onset profiles, with 110 and 44 AEs, respectively, emerging after 360 days of therapy. Brigatinib and Cabozantinib presented intermediate patterns, featuring both early- and late-onset events.

These findings suggest that early neurotoxicity monitoring is critical for Lorlatinib and Entretinib, while continued vigilance is necessary for long-term users of Crizotinib and Ceritinib.

### 3.5 Logistic regression analysis for fatal nervous system adverse events

**Figure 5:**
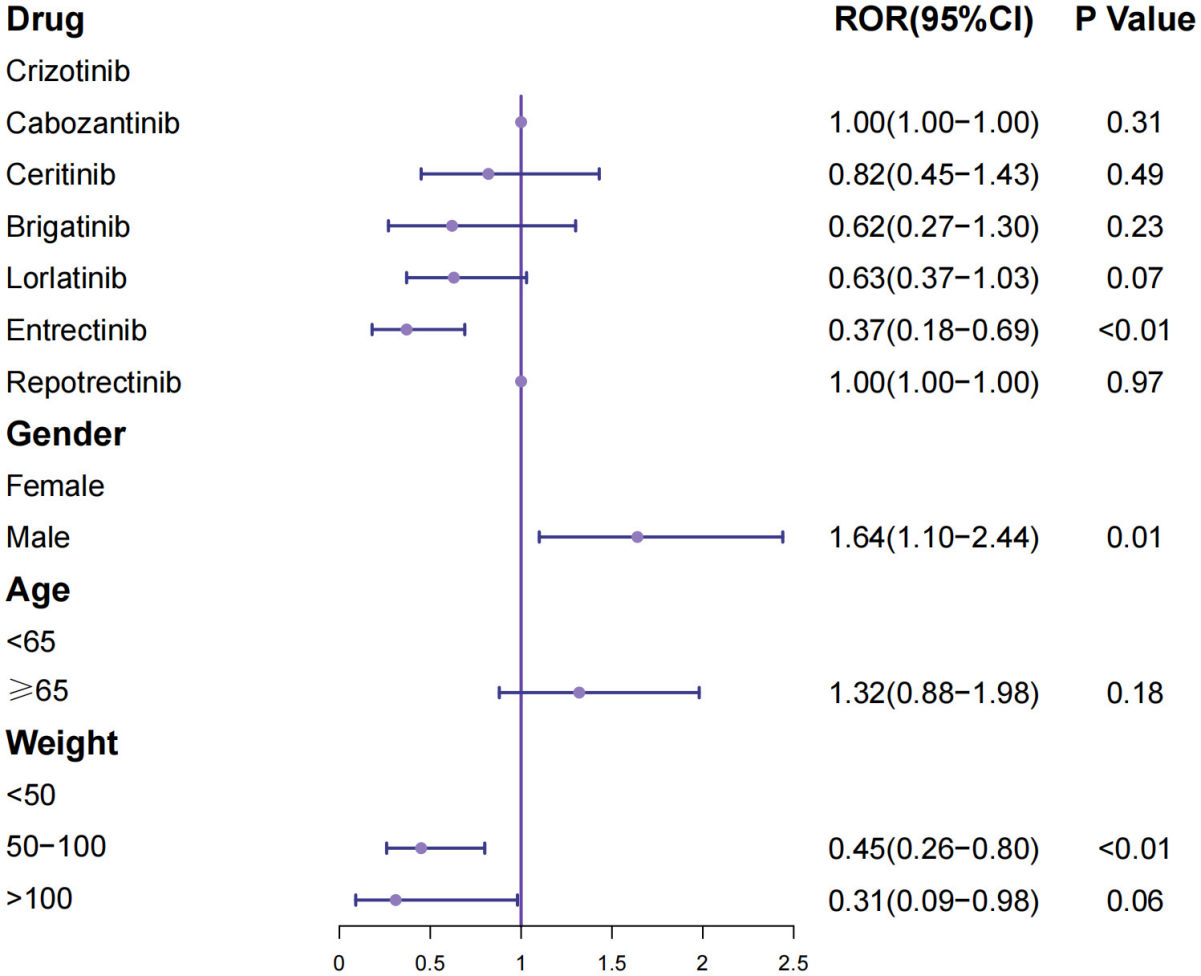
Multivariable logistic regression analysis of fatal nervous system adverse events associated with ROS1 inhibitors. Odds ratios (ORs) and 95% confidence intervals (CIs) are displayed for each drug, with Crizotinib serving as the reference group. Covariates include age group, sex, and body weight category.

Multivariable logistic regression was performed to identify predictors of fatal outcomes following nervous system AEs (Figure 5, Table 3). Compared with Crizotinib, Lorlatinib was associated with a significantly lower risk of death (OR 0.37, 95% CI 0.18–0.69, P <0.01). Male sex was independently associated with higher fatality risk (OR 1.64, 95% CI 1.10–2.44, *P* = 0.01), whereas older age (≥65 years) was protective (OR 0.45, 95% CI 0.26–0.80, *P* < 0.01).

**Table 3:** Logistic regression analysis for fatal nervous system adverse events.

**Characteristics**	**ROR (95%CI)**	***P* value**
**Crizotinib**		
Cabozantinib	1.00 (1.00–1.00)	0.31
Ceritinib	0.82 (0.45–1.43)	0.49
Brigatinib	0.62 (0.27–1.30)	0.23
Lorlatinib	0.63 (0.37–1.03)	0.07
Entrectinib	0.37 (0.18–0.69)	<0.01
Repotrectinib	1.00 (1.00–1.00)	0.97
**Gender**		
Female		
Male	1.64 (1.10–2.44)	0.01
**Age**		
<65		
≥65	1.32 (0.88–1.98)	0.18
**Weight**		
<50		
50–100	0.45 (0.26–0.80)	<0.01
>100	0.31 (0.09–0.98)	0.06

Overall, while certain ROS1 inhibitors exhibited stronger neurotoxicity signals, these events seldom resulted in fatal outcomes, indicating manageable yet clinically relevant CNS safety concerns.

## 4 Discussion

This pharmacovigilance analysis comprehensively compared the neurological safety profiles of seven ROS1 inhibitors using the FAERS database. We found that newer-generation inhibitors—Lorlatinib, Entretinib, and Reportinib—exhibited stronger and earlier-onset neurotoxicity signals, particularly involving cognitive impairment, dysgeusia, and dizziness, whereas earlier agents such as Crizotinib, Ceritinib, and Cabozantinib were associated with weaker or delayed neurological events. Despite their higher neurotoxicity signals, fatal neurological outcomes were uncommon, suggesting that most AEs are clinically manageable with appropriate monitoring and dose adjustments.

### 4.1 Comparison with previous studies

ROS1 inhibitors such as Crizotinib, Cabozantinib, and Ceritinib have demonstrated significant efficacy in the treatment of ROS1-positive tumors, providing valuable therapeutic options for patients with advanced non–small cell lung cancer (NSCLC). Nevertheless, their use is often accompanied by a range of adverse reactions, among which neurological complications have attracted increasing clinical attention. Understanding these neurological events is essential for evaluating the overall risk–benefit balance, optimizing therapeutic regimens, and improving patients' quality of life and prognosis.

The package insert for Crizotinib lists multiple-system adverse events, including neurologic symptoms such as headache (14, 15); however, both clinical studies and case reports indicate that the actual incidence of headache is much lower than reported. In a real-world observational study of ROS1-positive NSCLC, only a few patients developed mild, transient headache, which did not interrupt treatment. Our findings are consistent with these observations. Similarly, Cabozantinib has been linked to syncope in several large-scale clinical trials (e.g., METEOR, CELESTIAL), where the reported incidence ranged between 0% and 5%. In our analysis, syncope was also observed but demonstrated a lower signal strength compared with other adverse events. Although uncommon, this AE warrants clinical attention due to its potential severity.

By contrast, newer-generation inhibitors such as Lorlatinib, Entretinib, and Reportinib, designed to overcome resistance and enhance CNS penetration, displayed more prominent neurological AE signals in our study. This aligns with the results of major clinical trials such as CROWN and TRIDENT-1, in which dizziness, dysgeusia, paresthesia, and cognitive or mood alterations were among the most frequently reported treatment-related AEs. Collectively, these findings highlight that as molecular potency and CNS activity increase, neurological safety concerns also become more pronounced, necessitating careful post-marketing surveillance.

### 4.2 Mechanistic interpretation

The mechanisms underlying neurological AEs vary substantially among ROS1 inhibitors and are influenced by their molecular structure, kinase selectivity, and blood–brain barrier (BBB) permeability.

#### 4.2.1 Crizotinib and cabozantinib

Crizotinib exhibits relatively weak CNS penetration due to its larger molecular weight and moderate lipophilicity, leading to limited passive diffusion across the BBB. The primary targets of Crizotinib—ALK, ROS1, and MET—are minimally expressed in cerebellar and vestibular regions, which may explain the lower frequency of dizziness and headache observed in clinical practice (16, 17). Moreover, Crizotinib is a substrate of the P-glycoprotein (P-gp) transporter, which actively pumps the drug out of the brain and restricts intracerebral accumulation (18).

For Cabozantinib, syncope appears to arise from its potent inhibition of vascular endothelial growth factor receptors (VEGFRs). Suppression of VEGFR-mediated angiogenesis can lead to hypovolemia, endothelial dysfunction, and impaired nitric oxide release, collectively resulting in vasomotor instability, myocardial ischemia, and hypotension. Although the clinical incidence remains low, this mechanism may account for the sporadic but severe syncopal events observed in post-marketing data (19).

#### 4.2.2 Lorlatinib

Lorlatinib displayed the broadest and most intense neurotoxicity spectrum in this study, encompassing cognitive impairment, memory disorder, and speech abnormalities. Its macrocyclic amide structure substantially enhances BBB penetration, yielding cerebrospinal fluid (CSF)–to–plasma concentration ratios as high as 0.6–1.2—far exceeding those of Crizotinib [0.002–0.03; (11, 20)]. Such high intracerebral exposure can disrupt normal neuronal metabolism and signaling. Mechanistically, Lorlatinib may impair endothelial tight junctions by downregulating VEGF, TGF-β, and Claudin-5, thereby increasing BBB permeability (21). *In vitro* studies have further demonstrated that Lorlatinib can inhibit mitochondrial respiratory chain complexes, inducing excessive production of reactive oxygen species [ROS; (22)]. These oxidative insults lead to lipid peroxidation and membrane damage, ultimately impairing neuronal survival and synaptic transmission.

In hepatocyte models, Lorlatinib was shown to increase cholesterol ester and triglyceride accumulation. Under hyperlipidemic conditions, elevated free fatty acids can promote ROS generation in neurons, causing oxidative stress and neuroinflammation that affect cognitive, language, and emotional regulation pathways (23, 24). Our FAERS analysis also revealed non-negligible signals for peripheral neuropathy and paresthesia, consistent with previous reports showing a 39%−44% incidence of peripheral neuropathy in Lorlatinib-treated patients. These effects may result from interference with nerve growth factor (NGF)–related trophic signaling pathways, impairing neuronal maintenance and axonal function.

#### 4.2.3 Entretinib and reportinib

Both Entretinib and Reportinib are next-generation multitarget TKIs that inhibit ROS1 as well as tropomyosin receptor kinases (TRKA/B/C). Their shared adverse event, taste disorder, was strongly reflected in our data. This AE may be mediated by inhibition of TRKB (NTRK2), which plays a critical role in the differentiation and signal transduction of gustatory receptor cells (25, 26). Blocking TRKB disrupts taste signaling and receptor cell function, leading to dysgeusia. Because both agents exhibit strong BBB permeability, they can also influence CNS taste-processing centers, such as the nucleus tractus solitarius and insular cortex.

The temporal pattern of neurotoxicity among ROS1 inhibitors also reflects their pharmacologic diversity. Lorlatinib and Entretinib demonstrated early-onset neurotoxicity, typically within 30 days, due to rapid CNS distribution. Conversely, Crizotinib and Ceritinib showed delayed-onset neurotoxicity, likely attributable to cumulative exposure and slower CNS accumulation. Both are substrates of P-gp and BCRP transporters, which actively limit intracerebral concentrations and may explain their later manifestation of neurological AEs.

#### 4.3 Clinical implications

The distinct neurotoxicity profiles identified in this study have several practical implications.

First, clinicians should anticipate early neurological AEs, including cognitive impairment, dysgeusia, and dizziness, during the initial treatment phase with newer-generation inhibitors. Regular neurological evaluations within the first month of therapy can facilitate early detection and timely management. Second, for older agents such as Crizotinib and Ceritinib, long-term surveillance remains important since delayed-onset AEs may emerge after extended exposure due to gradual accumulation. Third, despite the higher frequency of neurological AEs in Lorlatinib, Entretinib, and Reportinib, most cases are mild, transient, and reversible. For instance, Entretinib-induced taste disturbances generally resolve within 4 months without discontinuation. Data from the BFAST trial confirmed that patients with CNS metastases could maintain therapy with dose modifications or supportive care without compromising overall survival (27, 28). Moreover, the low fatality rate associated with neurological AEs in our logistic regression analysis supports the notion that these toxicities are clinically manageable. Finally, understanding drug-specific neurotoxicity mechanisms may help clinicians tailor treatment based on patient comorbidities and neurological vulnerability. For example, Entretinib's strong CNS penetration but favorable safety in fatal outcomes makes it particularly suitable for patients with baseline CNS metastases who require intracranial control.

### 4.4 Limitations and future prospects

This study has inherent limitations stemming from the use of spontaneous reporting data.

First, the FAERS database depends on voluntary submissions from healthcare professionals and consumers, which may introduce underreporting, incomplete information, and selection bias. Second, FAERS data cannot establish a direct causal relationship between a drug and an AE; rather, it provides a signal of disproportional reporting that requires validation through controlled studies. Third, important variables such as treatment duration, dosage, disease severity, and concomitant medications were not available, limiting adjustment for confounders.

Future studies should integrate FAERS data with electronic health records, prescription databases, and prospective pharmacovigilance systems to improve causal inference and longitudinal tracking of neurological AEs. Experimental studies are also needed to elucidate molecular mechanisms, including TRK signaling and oxidative injury pathways, to better guide neuroprotective strategies during targeted therapy.

## 5 Conclusion

This study provides a comprehensive real-world assessment of neurological adverse events associated with ROS1 inhibitors. New-generation agents—Lorlatinib, Entretinib, and Reportinib—showed greater efficacy but stronger and earlier-onset neurotoxicity compared with earlier drugs such as Crizotinib and Ceritinib. The neurotoxicity of these inhibitors appears closely linked to their blood–brain barrier permeability, target selectivity, and pharmacokinetic properties. Clinically, individualized monitoring and early neurological assessment are essential to balance therapeutic benefits and safety, ensuring optimal outcomes for patients receiving ROS1-targeted therapy. Future pharmacovigilance and mechanistic studies are warranted to refine safety management and guide personalized treatment strategies.

## Data Availability

Publicly available datasets were analyzed in this study. This data can be found here: https://fis.fda.gov/extensions/FPD-QDE-FAERS/FPD-QDE-FAERS.html.
