# Supplementary material for: Neurological adverse events of ROS1 inhibitors for non-small cell lung cancer: data from the FDA adverse event reporting system
Source: Front Neurol. 2026 Jan 8;16:1691324. doi: 10.3389/fneur.2025.1691324 (PMC12823534; doi:10.3389/fneur.2025.1691324)
Supplement: Supplementary file 2 [file Table_2.docx]

| Drug | PT | N | ROR  (95%Cl) | PRR  (χ^2^) | EBGM  (EBGM05) | IC  (IC025) |
| --- | --- | --- | --- | --- | --- | --- |
| Crizotinib | Dysgeusia | 57 | 5.7 ( 4.29 - 7.58 ) | 5.68 ( 184.52 ) | 4.92 ( 3.88 ) | 2.3 ( 1.89 ) |
|  | Ageusia | 14 | 5.7 ( 3.21 - 10.1 ) | 5.69 ( 45.43 ) | 4.94 ( 3.06 ) | 2.3 ( 1.5 ) |
| Cabozantinib | Taste disorder | 4 | 8.99 ( 3.33 - 24.25 ) | 8.93 ( 27.67 ) | 8.78 ( 3.83 ) | 3.13 ( 1.82 ) |
|  | Syncope | 3 | 3.88 ( 1.24 - 12.11 ) | 3.86 ( 6.32 ) | 3.84 ( 1.48 ) | 1.94 ( 0.49 ) |
| Ceritinib | Central nervous system lesion | 13 | 6.2 ( 3.48 - 11.03 ) | 6.18 ( 50.34 ) | 5.62 ( 3.47 ) | 2.49 ( 1.67 ) |
|  | Epilepsy | 11 | 3.75 ( 2.03 - 6.93 ) | 3.75 ( 20.64 ) | 3.56 ( 2.13 ) | 1.83 ( 0.97 ) |
|  | Hemiplegia | 6 | 8.91 ( 3.74 - 21.23 ) | 8.9 ( 35.76 ) | 7.71 ( 3.73 ) | 2.95 ( 1.77 ) |
|  | Language disorder | 5 | 36.05 ( 11.44 - 113.62 ) | 36.01 ( 99.29 ) | 21.43 ( 8.2 ) | 4.42 ( 3 ) |
|  | Partial seizures | 4 | 8.07 ( 2.81 - 23.2 ) | 8.07 ( 21.35 ) | 7.09 ( 2.93 ) | 2.83 ( 1.43 ) |
|  | Paraplegia | 3 | 8.41 ( 2.48 - 28.55 ) | 8.4 ( 16.77 ) | 7.35 ( 2.64 ) | 2.88 ( 1.31 ) |
|  | Nerve compression | 3 | 10.09 ( 2.92 - 34.86 ) | 10.08 ( 20.46 ) | 8.57 ( 3.04 ) | 3.1 ( 1.51 ) |
| Brigatinib | Somnolence | 16 | 4.36 ( 2.63 - 7.25 ) | 4.35 ( 38.64 ) | 4.13 ( 2.7 ) | 2.05 ( 1.32 ) |
|  | Memory impairment | 14 | 3.88 ( 2.26 - 6.67 ) | 3.87 ( 28.13 ) | 3.71 ( 2.36 ) | 1.89 ( 1.12 ) |
|  | Subarachnoid haemorrhage | 3 | 8.2 ( 2.46 - 27.31 ) | 8.19 ( 16.75 ) | 7.36 ( 2.69 ) | 2.88 ( 1.33 ) |
| Lorlatinib | Cognitive disorder | 40 | 10.49 ( 7.44 - 14.79 ) | 10.4 ( 279.69 ) | 8.73 ( 6.55 ) | 3.13 ( 2.63 ) |
|  | Memory impairment | 40 | 11.22 ( 7.94 - 15.84 ) | 11.12 ( 299.52 ) | 9.22 ( 6.91 ) | 3.2 ( 2.71 ) |
|  | Neuropathy peripheral | 36 | 2.92 ( 2.08 - 4.09 ) | 2.9 ( 42.4 ) | 2.79 ( 2.1 ) | 1.48 ( 0.99 ) |
|  | Nervous system disorder | 18 | 6.58 ( 4.02 - 10.78 ) | 6.56 ( 74.67 ) | 5.89 ( 3.9 ) | 2.56 ( 1.85 ) |
|  | Speech disorder | 13 | 6.53 ( 3.66 - 11.66 ) | 6.51 ( 53.45 ) | 5.86 ( 3.6 ) | 2.55 ( 1.73 ) |
|  | Amnesia | 12 | 6.36 ( 3.48 - 11.62 ) | 6.34 ( 47.73 ) | 5.72 ( 3.45 ) | 2.52 ( 1.67 ) |
|  | Depressed level of consciousness | 10 | 4.26 ( 2.23 - 8.15 ) | 4.26 ( 22.9 ) | 3.99 ( 2.32 ) | 2 ( 1.09 ) |
|  | Dysarthria | 10 | 7.77 ( 3.98 - 15.17 ) | 7.76 ( 50.7 ) | 6.82 ( 3.9 ) | 2.77 ( 1.84 ) |
|  | Disturbance in attention | 9 | 10.09 ( 4.91 - 20.7 ) | 10.07 ( 60.78 ) | 8.5 ( 4.66 ) | 3.09 ( 2.09 ) |
|  | Aphasia | 8 | 5.07 ( 2.45 - 10.51 ) | 5.06 ( 23.61 ) | 4.68 ( 2.54 ) | 2.23 ( 1.21 ) |
|  | Carpal tunnel syndrome | 8 | 64.25 ( 22.28 - 185.25 ) | 64.12 ( 213.06 ) | 28.05 ( 11.57 ) | 4.81 ( 3.59 ) |
|  | Neurotoxicity | 8 | 4.94 ( 2.38 - 10.23 ) | 4.93 ( 22.76 ) | 4.57 ( 2.48 ) | 2.19 ( 1.18 ) |
|  | Cerebral disorder | 7 | 6.13 ( 2.79 - 13.47 ) | 6.12 ( 26.61 ) | 5.54 ( 2.87 ) | 2.47 ( 1.39 ) |
|  | Burning sensation | 6 | 5.45 ( 2.34 - 12.69 ) | 5.44 ( 19.56 ) | 4.99 ( 2.46 ) | 2.32 ( 1.17 ) |
|  | Mental impairment | 5 | 13.37 ( 4.96 - 36.04 ) | 13.36 ( 44.75 ) | 10.67 ( 4.66 ) | 3.42 ( 2.1 ) |
|  | Slow speech | 4 | 32.09 ( 9.05 - 113.77 ) | 32.06 ( 72.23 ) | 19.64 ( 6.81 ) | 4.3 ( 2.74 ) |
|  | Migraine | 4 | 5.66 ( 2.01 - 15.96 ) | 5.66 ( 13.73 ) | 5.17 ( 2.17 ) | 2.37 ( 1 ) |
|  | Brain fog | 4 | 17.5 ( 5.57 - 55 ) | 17.49 ( 45.6 ) | 13.09 ( 5.02 ) | 3.71 ( 2.23 ) |
|  | Leukoencephalopathy | 3 | 6.56 ( 1.96 - 21.93 ) | 6.56 ( 12.44 ) | 5.89 ( 2.15 ) | 2.56 ( 1.01 ) |
|  | Language disorder | 3 | 28.88 ( 6.9 - 120.87 ) | 28.86 ( 50.42 ) | 18.41 ( 5.56 ) | 4.2 ( 2.47 ) |
|  | Head discomfort | 3 | 8.49 ( 2.49 - 28.99 ) | 8.49 ( 16.84 ) | 7.36 ( 2.64 ) | 2.88 ( 1.31 ) |
| Entretinib | Dizziness | 51 | 11.11 ( 8.3 - 14.86 ) | 10.7 ( 416.4 ) | 9.97 ( 7.82 ) | 3.32 ( 2.89 ) |
|  | Taste disorder | 44 | 37.15 ( 26.5 - 52.09 ) | 35.89 ( 1174.97 ) | 28.44 ( 21.43 ) | 4.83 ( 4.35 ) |
|  | Cognitive disorder | 22 | 19.45 ( 12.39 - 30.52 ) | 19.12 ( 330.45 ) | 16.83 ( 11.55 ) | 4.07 ( 3.43 ) |
|  | Ataxia | 15 | 54.2 ( 29.67 - 99.01 ) | 53.57 ( 550.74 ) | 38.4 ( 23.2 ) | 5.26 ( 4.44 ) |
|  | Syncope | 10 | 7.04 ( 3.72 - 13.33 ) | 6.99 ( 48.82 ) | 6.69 ( 3.92 ) | 2.74 ( 1.84 ) |
|  | Balance disorder | 7 | 6.88 ( 3.21 - 14.75 ) | 6.85 ( 33.28 ) | 6.56 ( 3.47 ) | 2.71 ( 1.66 ) |
|  | Memory impairment | 6 | 4.45 ( 1.97 - 10.05 ) | 4.43 ( 15.43 ) | 4.32 ( 2.18 ) | 2.11 ( 1 ) |
|  | Dysgeusia | 6 | 6.47 ( 2.85 - 14.71 ) | 6.45 ( 26.34 ) | 6.19 ( 3.11 ) | 2.63 ( 1.51 ) |
|  | Dysarthria | 5 | 13.26 ( 5.28 - 33.31 ) | 13.21 ( 51.33 ) | 12.1 ( 5.6 ) | 3.6 ( 2.36 ) |
|  | Aphasia | 4 | 8.69 ( 3.15 - 23.93 ) | 8.66 ( 25.46 ) | 8.19 ( 3.51 ) | 3.03 ( 1.69 ) |
|  | Neuralgia | 3 | 7.64 ( 2.38 - 24.49 ) | 7.62 ( 16.33 ) | 7.26 ( 2.74 ) | 2.86 ( 1.36 ) |
|  | Dyslalia | 3 | 56.76 ( 14.66 - 219.75 ) | 56.63 ( 114.76 ) | 39.94 ( 12.87 ) | 5.32 ( 3.63 ) |
|  | Hyperaesthesia | 3 | 17.27 ( 5.18 - 57.6 ) | 17.23 ( 40.59 ) | 15.36 ( 5.61 ) | 3.94 ( 2.39 ) |
| Reportinib | Dizziness | 22 | 8.87 ( 5.75 - 13.67 ) | 8.34 ( 143.26 ) | 8.34 ( 5.81 ) | 3.06 ( 2.44 ) |
|  | Paraesthesia | 8 | 9.64 ( 4.78 - 19.44 ) | 9.43 ( 60.42 ) | 9.43 ( 5.24 ) | 3.24 ( 2.26 ) |
|  | Taste disorder | 7 | 74.66 ( 35.3 - 157.9 ) | 73.09 ( 497.66 ) | 73.06 ( 39.04 ) | 6.19 ( 5.16 ) |
|  | Balance disorder | 6 | 13.16 ( 5.87 - 29.5 ) | 12.93 ( 66.15 ) | 12.93 ( 6.58 ) | 3.69 ( 2.59 ) |
|  | Neuropathy peripheral | 6 | 12.33 ( 5.5 - 27.65 ) | 12.12 ( 61.32 ) | 12.12 ( 6.17 ) | 3.6 ( 2.5 ) |
|  | Dysgeusia | 3 | 7.42 ( 2.38 - 23.13 ) | 7.36 ( 16.52 ) | 7.36 ( 2.84 ) | 2.88 ( 1.43 ) |
|  | Nervous system disorder | 3 | 17.8 ( 5.71 - 55.48 ) | 17.65 ( 47.13 ) | 17.64 ( 6.82 ) | 4.14 ( 2.69 ) |
|  | Ataxia | 3 | 47.28 ( 15.17 - 147.37 ) | 46.85 ( 134.61 ) | 46.84 ( 18.09 ) | 5.55 ( 4.1 ) |
|  | Disturbance in attention | 3 | 10.29 ( 3.3 - 32.08 ) | 10.21 ( 24.94 ) | 10.21 ( 3.94 ) | 3.35 ( 1.9 ) |
