# Supplementary material for: Neurological adverse events of ROS1 inhibitors for non-small cell lung cancer: data from the FDA adverse event reporting system
Source: Front Neurol. 2026 Jan 8;16:1691324. doi: 10.3389/fneur.2025.1691324 (PMC12823534; doi:10.3389/fneur.2025.1691324)
Supplement: Supplementary file 3 [file Table_3.docx]

| Characteristics | ROR(95%CI) | P Value |
| --- | --- | --- |
| Crizotinib |  |  |
| Cabozantinib | 1.00(1.00-1.00) | 0.31 |
| Ceritinib | 0.82(0.45-1.43) | 0.49 |
| Brigatinib | 0.62(0.27-1.30) | 0.23 |
| Lorlatinib | 0.63(0.37-1.03) | 0.07 |
| Entrectinib | 0.37(0.18-0.69) | <0.01 |
| Repotrectinib | 1.00(1.00-1.00) | 0.97 |
| Gender |  |  |
| Female |  |  |
| Male | 1.64(1.10-2.44) | 0.01 |
| Age |  |  |
| <65 |  |  |
| ≥65 | 1.32(0.88-1.98) | 0.18 |
| Weight |  |  |
| ＜50 |  |  |
| 50-100 | 0.45(0.26-0.80) | <0.01 |
| >100 | 0.31(0.09-0.98) | 0.06 |
